# Differentials of fertility in North and South Gondar zones, northwest Ethiopia: A comparative cross-sectional study

**DOI:** 10.1186/1471-2458-8-397

**Published:** 2008-12-02

**Authors:** Getu Degu Alene, Alemayehu Worku

**Affiliations:** 1 School of Public Health, Faculty of Medicine, Addis Ababa University, Ethiopia

## Abstract

**Background:**

Ethiopia is one of the most densely populated countries in Africa with an estimated population of 77.1 million in mid-2007. Uncontrolled fertility has adversely influenced the socio-economic, demographic and environmental situations of the country. It is one of the largest and poorest countries that, even in the midst of crisis, has maintained high levels of fertility. This study was aimed at investigating the most important factors influencing fertility behavior in Northwest Ethiopia.

**Methods:**

A comparative cross-sectional study which included 2424 women aged 25 years and above was undertaken in the Amhara region of Northwest Ethiopia. The study subjects were grouped into high fertile and low fertile categories. There were 1011 and 1413 women in the high and low fertile groups, respectively. A multi-stage cluster sampling stratified by place of residence was employed to select the required study subjects. Both bivariate and multivariate logistic regression techniques were used to analyze the data.

**Results:**

Among the 25 variables considered in this study, only 9 of them were found significantly and independently associated with the level of fertility. Women with at least secondary education were at a lower risk of high fertility with OR = 0.37 (95% CI: 0.21 to 0.64) compared to those with no formal education. However, women with primary education did not show any significant difference when compared with the same baseline group. Age at first marriage was inversely associated with the number of children ever born alive. Place of residence, household expenditure, number of children who have died, attitude towards using contraceptives, women's knowledge on the safe period, and current marital status were the other variables that showed significant associations with the level of fertility.

**Conclusion:**

Female education beyond the primary level, reduced infant and child mortality, delayed marriage and correct knowledge on the safe period during the menstrual cycle were amongst the main factors that had a bearing on high fertility.

## Background

Today, to think of population is to think of sustainable growth. It is the phenomenon of growth that commands the attention of the current world. In this regard, it is usually said that less developed countries like Ethiopia could grow economically only if population growth is held in check [1-6]. It is now widely accepted that control of fertility, like the prevention of avoidable deaths, is a public health responsibility [1,2].

Ethiopia is one of the most densely populated countries in Africa (ranks second only to Nigeria) and suffers from direct and indirect population problems. Uncontrolled fertility has adversely influenced the socio-economic, demographic and environmental development of the country. Poverty, war and famine and associated low levels of education and health, weak infrastructure, and low agricultural and industrial production have exacerbated the problem of over population [4,7].

The demographic significance of Ethiopia when it comes to population growth on the African continent is very substantial. It is one of the largest and poorest countries that, even in the midst of crisis, has maintained high levels of fertility [8]. Its population has increased nearly seven times from 11.8 million at the beginning of the 20^th ^century to about 80 million today [4,9]. The annual rate of growth is estimated at 2.7% [9] and the population of the country will double itself in about 26 years if the present growth rate persists. A comparison of the total fertility rates (TFRs) calculated from the 2000 and 2005 Ethiopian Demographic and Health Surveys (DHS) shows little change for the country as a whole (5.5 births in 2000 versus 5.4 births in 2005) [10]. On the other hand, the contraceptive prevalence rates (CPRs) were 8.1% and 14.7% in 2000 and 2005, respectively [10] while the unmet needs for family planning during the same periods were reported as 36% and 34%) [10]. Currently, Ethiopia is not among the world's 15 most populous countries. However, it is estimated that it will take the 10^th ^rank with a projected population of 146 million by the year 2050 [11].

About 43% of the population of Ethiopia is below 15 years of age while only 3% is above 65 years. Even without taking into account many factors, such as, unemployment, under employment and a high prevalence of physical disability in the population, an economically active person (aged 15 to 64) in Ethiopia is obliged to carry nearly one dependent (inactive) individual. This shows that the age dependency burden is very high in this country [4,9,11].

As can be understood from the reports of World Health Organization [11,12], World Bank [13], the Ethiopian Demographic and Health Surveys (EDHS) and other similar reports [10,14,15], the status of this country in many of the major indicators is very poor. For example, the maternal mortality ratios of the country were 871 and 673 per 100,000 live births in 2000 and 2005, respectively [10,12]. The infant mortality rate as reported by the EDHS was 77 per 1000 live births in 2005 [10]. This is one of the highest in the world [4,10]. The life expectancy at birth is only 49 years [11] indicating one of the lowest survival rates on the planet. According to the 2007 Population Reference Bureau report, the life expectancy of the less developed countries excluding China is 66 years.

The situation in this country clearly illustrates the truism that demographic and developmental factors reinforce each other. High fertility and rapid population growth exert negative influences on economic and social development and low levels of economic and social development provide the climate favoring high fertility and hence rapid population growth. Thus under this situation, if one thinks about development in this country, then, one has to identify critical points through which to break into the vicious circle of such unfavorable phenomenon and mobilize sufficient energy and resources to that end.

The Amhara national regional state where the present study was undertaken is not an exception. It has a population of about 20 million [9]. The high plateaus of the region have been tremendously affected by uncontrolled population growth through out the ages. In this region, land degradation is becoming an acute problem. Population pressures and inappropriate subsistence farming practices contribute to soil impoverishment and erosion, rampant deforestation, overgrazing of common lands and misuse of agrochemicals.

The potential health service coverage of the region is about 27.7% (based on health stations and health centers) [9]. When this potential health service coverage is computed on the basis of health posts, health stations and health centers it is estimated to be 88.8% [9]. The infant mortality rate is 94 per 1000 live births and this rate is the highest among all regions of the country [9,10]. The annual rate of natural increase is estimated at 2.7%. According to the reports of the Central Statistical Agency (CSA, 2006), the total fertility rate and contraceptive prevalence rate (among married women) of this region were 5.1 and 15.7%, respectively [9,10].

The two zones of North and South Gondar where the present study was conducted consist of about 40% of the area of the entire Amhara region [16]. Nearly 28% of the population of the Amhara region lives in these zones [16]. A few fragmented studies carried out in these zones have documented the fact that most of the prevailing health indicators of these zones are similar to that of the entire region [10,15,17].

It is true that human fertility is a function of a variety of factors. These factors may vary from one place to the other depending on the specific conditions of the given area [10,18-25]. A proper understanding of these factors would be of paramount importance in tackling the problem of uncontrolled fertility which will pave the way for the improvement of the prevailing socio-economic problems of the country. In particular, it will have a substantial contribution in the improvement of the health status of women and children. It was therefore with this objective that this study which aimed at the identification of factors influencing fertility levels was undertaken in the Northwestern part of Ethiopia.

## Methods

This study was undertaken in North and South Gondar zones (formerly known as Begemider and Semein province) of the Amhara national regional state in Northwest Ethiopia from mid -October to mid-December, 2007. The Amhara region is one of the eleven regional states in Ethiopia. The two zones where this study was carried out constitute about 40% of the area of the Amhara region. One of these zones (North Gondar) borders with the Sudan. During the time of the study, North Gondar had twenty one *Weredas *(districts) with an estimated population of 2.9 million. Similarly, South Gondar was divided into eleven *Weredas *with a population of about 2.4 million.

The design of the study was a comparative cross sectional survey involving the high and low fertility groups. Women with number of children ever born alive greater than four were under the high fertility group. The other group which consisted of women with number of children less than or equal to four was taken as the low fertility group. A multi-stage cluster sampling technique was applied to select the required samples from the urban and rural areas of the study zones. From the twenty rural *Weredas *of North Gondar, five *Weredas *were selected by simple random sampling technique. Together with the town of Gondar, a total of six *Weredas *were considered in North Gondar. Similarly, from the ten rural *Weredas *of South Gondar, three *Weredas *were selected by simple random sampling technique. Together with the town of Debre Tabour, a total of four *Weredas *were considered in South Gondar. Consequently, a total of eight rural *Weredas *and two big urban centers were included in the present study. The two ancient towns of Gondar and Debre Tabour are the capital of North Gondar and South Gondar zones, respectively. Both towns (Gondar and Debre Tabour) served as the capital city of the whole country at different periods in the past. Currently, each of these towns has the status of *Woreda *administration.

Women aged 25 to 49 years residing in both urban and rural areas were considered. This group of women was taken for this particular study by taking account of the fact that women in the Amhara region are married at early ages and could have more than four children before they celebrate their twenty-fifth birthdays [10]. The minimum age was therefore set at 25 to give an equal chance for both the high and low fertile groups. After having the two broad divisions (urban/rural), multi-stage and other necessary random sampling schemes (depending on the specific condition of the given area) were used to capture the actual study subjects that would be included in the sample. About two-thirds of the study subjects were from rural *Kebeles *(localities) while the remaining one-third was from the urban centers. The urban centers, apart from the two big towns of Gondar and Debre Tabour, included the administrative centers (small towns) of the selected rural *Weredas*. A total of 32 clusters/localities were randomly selected and studied. Sixteen clusters were selected randomly from the rural areas and all women aged 25 to 49 were included in the study. Eight urban *kebeles *were taken by random selection from Gondar and Debre Tabour towns. The center of each of the eight Woreda administrations was also included. Women aged 25 to 49 were randomly selected from the big and small towns.

As the investigation was a comparative study, different sample sizes were calculated by taking account of the major determinant factors and using the STATCALC program of the EPI INFO statistical package. In this regard, a minimum detectable OR (Odds Ratio) of 2, a 5% level of significance (two-sided), a power of 90% and a one to one allocation ratio of high fertile group to low fertile group (n1: n2) were assumed. A design effect of 2 was considered and the computed sample sizes were multiplied by two. Moreover, some 5 percent was also added for non-response and other contingencies. This was done to increase the precision by reducing the sampling error.

Accordingly, by taking 83.5% (the proportion of non-users of family planning methods among women with ≤ 4 children) (10) and the above assumptions, a minimum sample size of 922 for each of the two groups was computed. This sample size was the highest when compared to the other sample sizes computed using different background characteristics of women. Although this minimum sample size was proposed at the design stage, the actual data collected from all clusters/*kebeles *included in the study were considerably higher than the anticipated one. A standardized structured questionnaire with closed and open ended questions was used to collect the required data. The questionnaire was partly prepared by taking account of the 2005 Demographic and Health Survey questions so as to make valid comparisons among the findings of similar studies.

The main outcome variable was the level of fertility categorized as high and low fertility groups. This classification of high fertility and low fertility was done based on the Population policy of Ethiopia which aims to have four children per woman and by taking account of similar studies undertaken in the country [4,18].

The predictor variables consisted of the basic demographic, socio-economic, contraceptive use, and other characteristics relating to women's sexual experience. The questionnaire was tested prior to collecting the actual data and amendments were made depending on the results of the pre-test.

Data collection was carried out by twenty health professionals (health officers, nurses and environmental health technicians) who were given a three-day intensive training with practical exercises. Five health officers/sanitarians were assigned to supervise the data collection process and the overall coordination was handled by the investigators of the research project. Various appropriate measures were taken to ensure the quality of the data that were collected from the proposed study areas. In this regard, one of the duties of the supervisors was to randomly select 5% of the already surveyed households for cross-checking and ensure the reliability of the collected data. Incomplete questionnaires were filled by making re-visits while on fieldwork. In fact, the data collectors were informed about the strict supervision and the cross-checking procedure that would take place during data collection.

Data entry into the computer was carried out using the Statistical Package for Social Sciences (SPSS) for Windows version 11. Data analysis was also undertaken using the same statistical package. The main statistical method applied was logistic regression (unconditional) and both the classical bivariate and multivariate analyses were considered. The unadjusted (crude) and adjusted Odds ratios together with their corresponding 95% confidence intervals were computed. In this paper, the term likelihood is used to mean odds. A P-value ≤ 0.05 was considered statistically significant in this study.

Efforts were made to assess whether the necessary assumptions for the application of multiple logistic regression were fulfilled. In this regard, the Hosmer and Lemeshow's goodness-of-fit test was considered. This statistic is computed as the Pearson chi-square from the contingency table of observed frequencies and expected frequencies. A good fit as measured by Hosmer and Lemeshow's test will yield a large P-value.

Ethical clearance was obtained from the School of Public Health and the Faculty of Medicine of Addis Ababa University. Written consent was obtained from the responsible Zone and *Woreda *government organizations by explaining the objectives of the study. Verbal consent was obtained from each study subject included in the study.

## Results

A total of 1011 women in high fertile group and 1413 women in low fertile group were included in this study. About 68% (that is, 1642 out of 2424) of the participants were from the rural areas of North and South Gondar zones. Over three-fourths of the women were housewives. Nearly 92% of them were from families with monthly household income of less than US $110. Orthodox Christianity was the main religion for about 93% of the respondents followed by Muslims which constituted nearly 6.6% of the overall study subjects. This study revealed the very fact that 22.7% of the responding women aged 25 to 49 had at least a primary education. Only 9.6% the entire women of this female population reported that they had high school or college education. It was learned from the present study that the mean number of children per woman in the high fertile group was 6.5 (median = 6.0) while it was 2.6 (median = 3.0) in the low fertile group. The corresponding standard deviations were also computed as 1.5 and 1.2 in the high and low fertile groups, respectively.

As can be noted from the findings of the bivariate analyses (Tables 1 and 2), six of the twenty five variables did not show a significant association with the outcome variable at a 5% level of significance. In this regard, religion, ethnic group, treatment seeking behavior when a family member gets sick and the observation of the respondent towards population increase within her dwelling area were not significant at a 0.3 level of significance and were excluded from further analyses. In fact, the corresponding P-values for each of these variables were greater than 0.48. The other two predictor variables (choice of the respondent regarding the sex of her first child and zone) which fulfilled the minimum requirement for further assessment were considered and entered into the multivariate logistic regression model. Each of these predictor variables showed statistical significance at a 0.3 level of significance (Tables 1 and 2). Consequently, the multivariate logistic regression analysis which controls the undesirable effects of confounding variables was used by taking all the twenty one covariates (predictor variables) into account simultaneously. The backward stepwise regression which controls the problem of multicollinearity was employed and only nine of the most contributing factors remained to be significantly and independently associated with the level of fertility (Table 3). The same findings were also obtained using the forward stepwise regression.

**Table 1:** Results of separately regressing fertility levels (high versus low) on each demographic and socio-economic explanatory variable, North and South Gondar zones, northwest Ethiopia, 2007 (Bivariate analyses)

**Explanatory variable**	**Fertility level**		**Odds Ratio (crude)**	**95% Confidence Interval**	
			
	**High**	**Low**		**Lower**	**Upper**
Place of residence					
Big towns	84	389	1.00		
Small towns	79	230	1.59	1.12	2.25
Rural areas	848	794	4.95	3.83	6.38
					
Current marital status					
Married	889	1088	2.18	1.74	2.73
Others	122	325	1.00		
					
Educational level					
No education	884	989	1.00		
Primary	104	214	0.54	0.42	0.70
Secondary and above	23	210	0.12	0.08	0.19
					
Source of drinking water					
Piped	349	803	1.00		
Protected well/spring	209	209	2.30	1.83	2.89
Unprotected well/spring	250	179	3.21	2.56	4.04
River water	203	222	2.10	1.68	2.64
					
Religion *					
Orthodox Christian	945	1312	1.00		
Moslem	64	95	0.94	0.67	1.30
					
Has radio					
Yes, functional	369	685	1.00		
Yes, non-functional	122	137	1.65	1.26	2.18
No	520	591	1.63	1.37	1.94
					
Ethnicity *					
Amhara	974	1359	1.00		
Others	37	54	0.96	0.62	1.46
					
Number of children who have died					
0	513	1235	1.00		
1–2 children	382	166	5.54	4.49	6.83
3 and above children	116	12	23.12	12.66	42.20
					
Occupation of the respondent					
Working (civil servant + trader + student)	28	151	1.00		
Farmer	117	108	5.84	3.61	9.45
Housewife	832	992	4.52	2.99	6.84
Others	34	162	1.13	0.66	1.96
Main roof material					
Thatch/bamboo/reed	382	462	1.00		
Corrugated iron	629	951	0.80	0.68	0.95
					
When one of the family members gets sick, place where the sick person is taken first: *					
Modern health institutions	982	1379	1.00		
Traditional healers/holly water/etc.	29	34	1.20	0.73	1.98
					
Decision on own health care					
The woman herself	253	481	1.00		
Husband	119	96	2.36	1.73	3.21
Both	637	806	1.50	1.25	1.81
Other	2	30	0.13	0.03	0.54
					
Monthly household expenditure					
≤ 320 Eth Birr	256	556	1.00		
321 – 500 Eth Birr	309	487	1.38	1.12	1.69
501 – 999 Eth Birr	327	292	2.43	1.96	3.02
≥ 1000 Eth Birr	119	78	3.31	2.40	4.57
					
Is there any visible population increase in your (respondent's) dwelling area? *					
Yes	971	1353	1.00	0.62	1.40
No	40	60	0.93		
					
Choice of the respondent regarding the sex of her first child					
Male	563	726	1.00	0.67	0.97
Female	293	470	0.80	0.73	1.16
Any sex	155	217	0.92		
					
Total number of children born alive from the mother of the respondent					
1 – 4 children	150	321	1.00		
5 – 9 children	650	929	1.50	1.20	1.86
10 and above	211	162	2.79	2.10	3.69
					
Do you approve wife beating by the husband for various reasons?					
Yes	735	847	1.78	1.49	2.12
No	276	566	1.00		
					
Zone					
North Gondar	597	877	1.00		
South Gondar	414	536	1.13	0.96	1.34

* shows non-significance at a 0.3 level of significance.

**Table 2:** Results of separately regressing fertility levels (high versus low) on each explanatory variable relating to women's sexual behaviour and use of contraceptives, North and South Gondar zones, northwest Ethiopia, 2007 (Bivariate analyses)

**Explanatory variable**	**Fertility level**		**Odds Ratio (crude)**	**95% Confidence Interval**	
			
	**High**	**Low**		**Lower**	**Upper**
Age at first marriage*					
< 15 years (ref. category)	635	553	1.00		
15 – 19 years	353	638	0.48	0.41	0.58
20 and above	23	200	0.10	0.06	0.16
					
Age at first sexual intercourse**					
< 15 years (ref. category)	512	406	1.00		
15 – 19 years	466	788	0.47	0.39	0.56
20 and above	33	204	0.13	0.09	0.19
					
Current use of contraceptives**					
Yes	220	356	1.00		
No	791	1042	1.23	1.02	1.49
					
Attitude towards using contraceptives in the future					
Yes	552	860	1.00		
No	459	553	1.29	1.10	1.52
					
Should female circumcision continue?					
Yes	107	103	1.00		
No	904	1310	0.66	0.50	0.88
					
Knowledge of the respondent regarding the period of pregnancy					
Correct	92	274	1.00		
Wrong	919	1139	2.40	1.87	3.09
					
If you want, can you find condoms easily?**					
Yes	188	448	1.00		
No	823	950	2.06	1.70	2.51
	-	15	-	-	-

* Women who were never married were excluded from the analysis

** Women with no experience of sexual intercourse were omitted from the analysis

**Table 3:** Results from the multivariate analysis – adjusted for demographic, socio-economic and reproductive variables, North and South Gondar zones, northwest Ethiopia, 2007

**Explanatory variable**	**Fertility level**		**OR (adjusted)**	**95% C. I.**		**P-value**
				
	**High**	**Low**		**Lower**	**Upper**	
Place of residence						< 0.001*
Big towns	84	389	1.00			
Small towns	79	230	1.27	0.86	1.90	0.235
Rural areas	848	794	2.79	2.01	3.87	< 0.001
						
Age at first marriage						< 0.001*
< 15 years	635	553	1.00			
15 – 19 years	353	638	0.65	0.53	0.80	< 0.001
20 and above	23	200	0.21	0.13	0.36	< 0.001
						
Current marital status						
Married	889	1088	1.62	1.20	2.19	0.002
Others	122	325	1.00			
						
Educational level						0.002
No education	884	989	1.00			
Primary	104	214	0.92	0.67	1.27	0.613
Secondary and above	23	210	0.37	0.21	0.64	< 0.001
						
Monthly household expenditure						< 0.001*
≤ 320 Eth Birr	256	556	1.00			
321 – 500 Eth Birr	309	487	1.48	1.16	1.88	0.001
501 – 999 Eth Birr	327	292	3.39	2.60	4.43	< 0.001
≥ 1000 Eth Birr	119	78	6.97	4.54	10.71	< 0.001
						
Number of children who have died						< 0.001*
none	513	1235	1.00			
1–2 children	382	166	4.31	3.42	5.42	< 0.001
3 and above children	116	12	19.87	10.45	37.77	< 0.001
						
Knowledge of the respondent regarding the period of pregnancy						
Correct	92	274	1.00			
Wrong	919	1139	1.42	1.04	1.93	0.027
						
Attitude towards using contraceptives in the future						
Yes	552	860	1.00			
No	459	553	1.26	1.02	1.55	0.030
						
Total number of children born alive from the mother of the respondent						
1 – 4 children	150	321	1.00			
5 – 9 children	650	929	1.27	0.97	1.64	0.079
10 and above	211	162	2.16	1.55	3.03	< 0.001

* For variables having more than two categories, the overall significance is given by their corresponding P – values N.B. The combined contribution of the rest 12 variables was very minimal (LRT = 19.67, (df = 20), P > > 0.20). This justifies the dropping of these variables so as to have a more parsimonious model that works just as the full model.

Accordingly, women living in rural villages were 2.8 times more likely to have high fertility as compared to those living in big towns of Gondar and Debre Tabour (OR = 2.79, 95%CI: 2.01, 3.87). However, the difference in the level of fertility between women of the big and small towns fell short of statistical significance (OR = 1.27, 95% CI: 0.86, 1.90).

The investigation made on whether women who were currently married had a different fertility experience from those not currently in such a union; currently married women were 62% more likely to have a high fertility as compared with unmarried women (OR = 1.62, 95%CI: 1.20, 2.19).

Age at first marriage was the other independent variable which had a significant impact on the fertility status of women of the study areas. There had been a progressive decrease in the number of children with an increase of age at first marriage. The corresponding Odds Ratios for women 15 to 19 and 20 and above were 0.65 and 0.21, respectively, indicating the inverse association of late age at first marriage and the level of women's fertility.

The analysis made on the knowledge of women about the fertile period between the menstrual cycles showed a significant difference between high and low fertility groups. Those women who didn't have the correct knowledge were 1.42 times more likely to undergo the risk of high fertility (OR = 1.42, 95%CI:1.04, 1.93).

In this study it was learned that the educational status of women had an overall significant effect on the number of children that women would have in their life time (P = 0.002). In this regard, although the overall trend observed was quite significant, it was only women with a high school or above education that ended up with a low risk of having high fertility compared to those without modern education (OR = 0.37, 95% CI: 0.21, 0.64). On the other hand, those women with a primary education didn't have a significant difference from those with no formal education (OR = 0.92, 95% CI: 0.67, 1.27).

History of child death was found to have had a very high association with an increased number of children. As the number of children who had died increased, there appeared an increasing trend in the number of children ever born alive. Mothers who had lost 1 to 2 and three and more children were about 4 and 20 times more likely to have resulted in higher fertility with 95% confidence intervals of (3.42, 5.42) and (10.45, 37.77), respectively.

Current use of contraceptives which showed a significant association with the fertility level of women in the bivariate analysis (P = 0.04) turned out to be marginally significant (P = 0.08) in the multivariate analysis. There was some evidence (at an α-value of 0.1) that current users were at a reduced risk of high fertility compared with non-users (OR = 0.8, 95%CI: 0.58, 1.04). On the other hand, the attitude of women towards future use of contraceptives remained to be significantly associated with the outcome variable even after controlling for many other variables (P = 0.03). Accordingly, women who reported not to use any modern contraceptives in the future were 1.26 times more likely to face the risk of high fertility.

Monthly household expenditure showed a direct association with the number of children ever born alive. As can be seen from Table 3, with the increase of household expenditure there appeared a corresponding progressive increase in the number of children ever born alive. A similar phenomenon was also observed when the number of children ever born alive from the respondent's mother was compared with the number of children of her own.

The majority of the variables which showed significant associations with the level of fertility in the bivariate analyses could not persist in having such associations in the multivariate analyses. Such variables were: source of drinking water, possession of radio, main roof material, age at first sexual intercourse, decision on own health care, preference of sex of first child, occupation, wife beating, easy access to condoms, attitude of women towards female circumcision, and zone.

Apart from identifying the important contributing variables that affect fertility either positively or negatively, this study had also explored the conceived reasons why some women were in favor of a large number of children. Slightly greater than a third of the responding subjects approved the advantages of having five or more children and their main reason for such attitude was the economical benefits that might be obtained from a large number of children. Furthermore, the type of preference they had towards the sex of the first child and their reasons for such preferences were also examined. In this regard, about 53% and 32% of the women were in favor of male and female children, respectively.

The assessment made whether the required assumptions for the application of multiple logistic regression was fulfilled showed that the present parsimonious model adequately fits the data as P = 0.886.

## Discussion and conclusion

Ethiopia is a country which has been ravaged by both man made and natural disasters which resulted in the emergence of a core group of 5 to 7 million people who are in chronic need of food aid from the international community each year [26,27]. The history of the country over the last thirty years has been associated with famine and drought. In spite of such gloomy experience in which significant portions of its people are living under extreme poverty, the population of Ethiopia continues to grow rapidly by an estimated 2 million people annually [26]. Under these circumstances, achieving important national goals, such as, food self sufficiency, accessibility of citizens to health services, increasing employment opportunities, reducing underemployment and improving housing conditions are proving to be exceedingly difficult under a scenario of uncontrolled rapid population growth [14,15,22,24,27,28].

In order to effectively tackle the uncontrolled population growth and its associated problems in Ethiopia in general and in the present study areas in particular, there appears a need to investigate the contribution of a number of factors influencing fertility. Accordingly, this study has attempted to look into differentials of fertility in a typical rural and urban set ups by incorporating as many risk factors as possible.

Education as a whole has a negative effect on uncontrolled birth. This was true even when the data were re-analyzed by adjusting for many other demographic, reproductive and socio-economic variables. Those women who had at least a high school education showed nearly a two-third reduction in fertility compared to women with no education (OR = 0.37, 95% CI: 0.21, 0.64). This finding is compatible with the results of many other similar studies [26,29-31]. However, in this study, there was no evidence that primary education had an effect on high fertility (OR = 0.92, 95% CI: 0.67, 1.27). A study undertaken in the Southern part of Ethiopia revealed a situation in which women with a few years of schooling had higher level of fertility compared with those women with no formal schooling [14]. A similar finding was also reported by Caldwell [32].

Girls who quit schooling at the primary level would soon be overburdened by the existing rural cultures and get married. For example, in Gondar zone alone, there were 21,875 students (about 50% of these were females) who discontinued their primary education in the last one year [33]. This requires the development of an enabling condition that would reduce the number of female students who fail to continue their education due to one or another reason. In this regard, the Ethiopian education policy should encourage female students to complete at least the first cycle of their high school education besides improving the quality of the current primary education. It is to be noted that education is instrumental not only to reduce uncontrolled fertility, but also to enhance many other developmental activities [34,35].

An increase in child death has tremendously affected fertility. As the number of children died increased women were exposed to a higher risk of having more and more uncontrolled fertility. This requires a concerted effort in reducing infant and child mortality by putting in place strong measures, such as, vaccination, provision of safe water, proper schooling, etc. Reducing infant and child mortality, apart from giving mothers (parents) confidence to limit the number of children they would like to have, will also increase the life expectancies of both men and women to a greater extent. Moreover, women will have the opportunity to be engaged in many other activities that would ultimately lead them to gain a greater empowerment. A similar finding was obtained in the Butajira study of Central Ethiopia [36].

An increase in the average age at first marriage has an adverse effect on high fertility. Those women who get married at early age will be exposed to an early sexual intercourse which in turn leads to too many teen age pregnancies. Apart from the negative impact it poses on women's health, this culture of early marriage has a greater likelihood of having a lot of children eventually. Similar findings were documented in a number other studies [18,30]. The minimum age of marriage for girls and boys is set at 18 years in Ethiopia in general and in the Amhara region in Particular [37]. However, this has little impact on the traditional societies, where getting married and having children are considered the only proper roles of women [15,38,39]. The experience of Tunisia could be of paramount importance in this regard. In Tunisia, the steady decline in fertility observed during the last two decades has been attributed to joint action to raise the marriage age and to promote the use of contraception during the past three decades. The success of the fertility transition in Tunisia can also be explained by the fact that the country's population policy is not only well planned, but is also backed by relevant legislation and by political will at the highest level [3].

Marital status (currently married vs. those not in unions) had an impact on fertility. The married ones were 1.62 times more likely to be under the risk of high fertility compared to those women who were not in unions. This sounds true in such communities where the use of family planning and female education are at the lowest level. Moreover, the culture of the population does not encourage births out of marriage (mostly true in the Amhara region).

In the present study, current use of contraceptives was only marginally significant. There was not enough evidence at the 5% level of significance to reject the null hypothesis of no significant association between current contraceptive use and fertility level. This indicates that both groups (high and low fertile women) were about the same in using contraceptives (22% vs. 25%). Similar findings were documented in the Gondar and Butajira studies [15,18]. On the other hand, the attitude of women to use contraceptives in the future is very promising. This tendency of women to use contraceptives should be well taken by the concerned bodies (both governmental organizations and NGOs) and much effort should be exerted in facilitating the provision of the required service to the population in need.

The two zonal towns of Gondar and Debre Tabour (big towns) were at a reduced risk of high fertility. Women in the rural areas were nearly 2.8 times more likely to have had high fertility compared to women of these towns. This is generally true that girls residing in big towns like Debre Tabour and Gondar will stay longer in schools thereby delaying the time for marital engagement. On the other hand, the centers of the district administrations, which were called small towns in this study, were not significantly different from those of the big towns when it comes to fertility. That is, this study had revealed a similar fertility experience between these two dwelling areas. This could probably be due to their similarities in the modes of living. Moreover, the demanding living conditions in both big and small towns would not allow couples to have a large family.

Monthly household expenditure was used as a proxy indicator of monthly household income. Unlike the findings from the Butajira study which was conducted in central Ethiopia [18], monthly household income was positively associated with fertility in the present study. As monthly income increased, there appeared a progressive increase in the number of children ever born alive. This finding and the attitude of a few women in the present study areas who gave approval for rich people to have many more children should deserve special attention. The efforts of the country towards the eradication of poverty, if followed by such attitudes and practices, could be seriously hampered.

The knowledge of women on the safe period during the menstrual cycle was one of the factors that had a bearing on the level of fertility. Those women who were unable to report the safe period were 1.42 times more likely to be under the risk of high fertility. The great majority of the women (about 85%) lacking the correct knowledge about the safe period is indicator of the prevailing fact on the ground. Currently, the health service extension workers who are given a one year training are assigned in all rural *Kebeles *of the Amhara region. They make frequent visits to all households of their catchment areas to teach, persuade and motivate the mothers to adopt the new health actions. Moreover, they are expected to give the required health services including the provision of contraceptives. Although this endeavor has been in place since 2005 there are still a lot of women who have not acquired the correct knowledge about the safe period. It is therefore important to make periodic assessment whether these community-based health service extension workers are performing in line with the set programs.

In conclusion, female education beyond the primary level, reduced infant and child mortality, delayed marriage and correct knowledge on safe period during the menstrual cycle were among the most contributing factors in reducing high fertility. In particular, the enhancement of female education beyond the primary level is the most important agent of change in women's access to power and control over resources, as well as in demographic conditions. In this regard, the efforts of the Ethiopian government to achieve a universal primary education among the Ethiopian nationals should be encouraged and further steps that would enable female students to pursue their secondary and college education should be sought.

It is true that the absence of a theoretically-grounded conceptual framework for the construction of the regression models could be considered a limitation. However, it is believed that the unavailability of such a framework would not markedly affect the findings of the present study. In light of this, the following recommendations are forwarded.

♣ The education policy of the country should devise mechanisms that improve the quality of primary education. Female students should be encouraged to complete at least the first cycle of their secondary school education. Special arrangements need to be developed and be in place to reduce the number of student dropouts particularly female ones. The most important issues relating to reproductive health need to be incorporated into the primary school curricula.

♣ Reduction of infant and child mortality by putting in place strong measures, such as, vaccination, provision of safe water, etc. Incorporating the basic disease prevention methods in primary schools could also be considered.

♣ In Ethiopia in general and in the present study areas in particular, marriage is the destiny of nearly all people. The legal age at first marriage in Ethiopia is 18 years. However, this minimum age at first marriage is not implemented particularly in the present study areas. Hence, all responsible bodies including the remotest *Kebele *administrations should be in a position to ensure the practicability of this marriage law. Those couples (parents) who break this marriage law should be fined.

♣ It was understood from the present study that there was some evidence regarding the negative influence of current use of contraceptives on high fertility. In this regard, the family planning programs of the region should be strengthened to the extent that they could play significant roles in bringing down the prevailing high fertility. Accordingly, the main stakeholders should exert maximum efforts to make the method of choice available and accessible to the users.

## Competing interests

The authors declare that they have no competing interests.

## Authors' contributions

GDA wrote the proposal, participated in data collection, analyzed the data and drafted the paper. AW approved the proposal with some revisions, participated in data collection, commented on the analysis and improved the first draft. GDA and AW revised subsequent drafts of the paper. All authors read and approved the final manuscript.

## Pre-publication history

The pre-publication history for this paper can be accessed here:


